# Investigating Transcranial Direct Current Stimulation (TDCS) in obsessive compulsive disorder (OCD): a double-blind, sham-controlled, cross-over randomised trial

**DOI:** 10.1192/bjo.2021.96

**Published:** 2021-06-18

**Authors:** Eduardo Cinosi, David Baldwin, Tim Gale, Matt Garner, Natalie Hall, Daniel Meron, Nick Sireau, David Wellsted, Solange Wyatt, Naomi A Fineberg

**Affiliations:** 1 Hertfordshire Partnership University NHS Foundation Trust, University of Hertfordshire; 2 University of Southampton; 3 University of Hertfordshire; 4 University of Southampton, Somerset Partnership NHS Foundation Trust; 5 ORCHARD- Advancing Global OCD Research Charity; 6 Hertfordshire Partnership University NHS Foundation Trust, University of Hertfordshire, University of Cambridge

## Abstract

**Aims:**

Obsessive Compulsive Disorder is a disabling and difficult-to-treat condition, new treatment options are needed to improve health outcomes. Transcranial Direct Current Stimulation, a non-invasive form of neurostimulation, has shown positive results in a small number of studies as a safe and potentially efficacious treatment for OCD. There nevertheless remains uncertainty about the optimal stimulation protocol, magnitude and duration of effect, acceptability, tolerability and practicality of applying tDCS clinical settings. As existing data are inadequate to support a full-scale trial, we will deliver a feasibility study to address key research questions and knowledge gaps to enable the design and the development of the most efficient, cost effective, definitive trial.

**Method:**

We designed Feasibility And Acceptability Of Transcranial Stimulation In Obsessive Compulsive Symptoms (FEATSOCS), a double-blind, sham-controlled, cross-over randomised multicentre study in 25 adults with OCD. We will stimulate the two most promising cortical sites, the orbitofrontal cortex (OFC) and the supplementary motor area (SMA). Each participant will receive three courses of tDCS (SMA, OFC and sham), randomly allocated, given in counterbalanced order. Each course comprises four 20 minutes-stimulations, delivered over two consecutive days, separated by at least four weeks’ washout period. Blinded raters will regularly assess clinical outcomes before, during and up to four weeks after stimulation using validated scales. We will include relevant neurocognitive tasks, testing cognitive flexibility, motor disinhibition, cooperation and habit learning.

**Result:**

FEATSOCS trial is currently underway and recruiting. Owing to the impact of COVID-19, a recruitment extension has been granted. At the study end, we will analyse the feasibility outcomes, magnitude of the effect of the interventions on OCD symptoms alongside the standard deviation of the outcome measure to estimate effect size, and determine the optimal stimulation target. We will also measure the duration of the effect of stimulation, to provide information on spacing treatments efficiently. We will evaluate the usefulness and limitations of specific neurocognitive tests to determine a definitive test battery. Qualitative data will be collected from participants to better understand their experience of taking part in a tDCS intervention, the impact on their overall quality of life and their views on the potential of tDCS as home based-intervention.

**Conclusion:**

Further evidence is needed to establish whether tDCS could join the treatment armamentarium of OCD. The clinical outcomes in FEATSOCS will enable to further refine the methodology to ensure optimal efficiency in terms of both delivering and assessing the tDCS in OCD in a full scale trial.

The funder for this study is the National Institute for Health Research Programme, Research for Patient Benefit (RfPB) [Ref. no PB-PG-1216-20005]. Extra funding to allow study extension was provided by Orchard OCD. This study has received full ethics committee approval and protocol amendments approval form the Cambridge and Hertfordshire NHS Research Ethics Committee, IRAS Project ID 254507, REC ref: 19/EE/0046.

